# Sympathetic modulation as a goal of antihypertensive treatment: from drugs to devices

**DOI:** 10.1097/HJH.0000000000003538

**Published:** 2023-09-04

**Authors:** Guido Grassi

**Affiliations:** Clinica Medica, Department of Medicine and Surgery, University of Milano-Bicocca, Milan, Italy

**Keywords:** bariatric surgery, baroreceptor activation therapy, combination drug treatment, continuous positive airway pressure, dietary salt restriction, dietary weight reduction, monotherapy, physical exercise training, refractory hypertension, renal nerves ablation, residual risk, sympathetic nervous system

## Abstract

The present study aims to examine the effects of nonpharmacological, pharmacological and devices-based treatment on hypertension-related sympathetic overactivity. This will be done by analyzing the results of different published studies, in which sympathetic activity has been assessed via indirect or direct techniques. After examining the rationale for sympathomodulatory interventions in antihypertensive treatment, the study will discuss the methodological intrinsic limitations of the studies aimed at assessing different therapeutic interventions. The core of the study will be then focused on the effects of nonpharmacological (dietary restriction of sodium intake, physical exercise training, weight reduction), pharmacological (monotherapy, combination drug treatment, new drugs such as sodium glucose co-transport protein-2 inhibitors and angiotensin receptor neprilysin inhibitors), as well as devices-based interventions (renal sympathetic nerves ablation and carotid baroreceptor activation therapy) on the hypertension-related sympathetic overdrive. Finally, the areas worthy of future research as well as the debated issues in the field will be highlighted.

## INTRODUCTION

**FIGURE 1:**
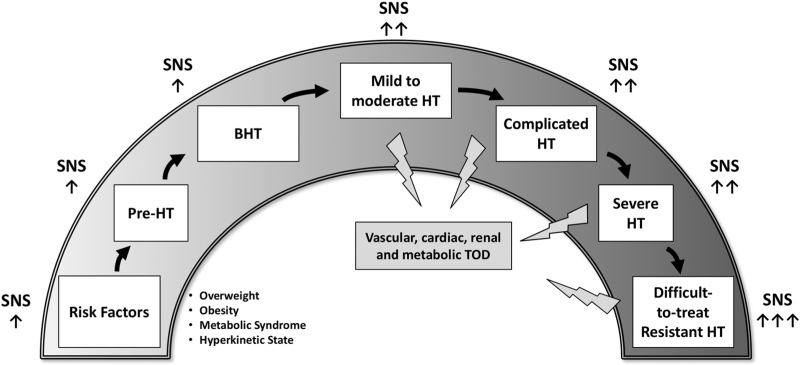
Chain of events which participate at the cardiovascular continuum, favoring the progression of the disease, the occurrence of cardiovascular complications and the development of the hypertension-related end organ damage. HT, hypertension; SNS, sympathetic nervous system; TOD, target organ damage. ↑: increase; ↑↑: marked increase; ↑↑↑: very marked increase.

Original studies, frequently included in meta-analyses, based on evaluation of direct as well as on indirect markers of sympathetic cardiovascular drive, have conclusively shown that neuroadrenergic influences to the heart and the peripheral circulation are markedly activated in hypertensive patients, the resulting sympathetic overdrive representing a hallmark of the hypertensive disease [1–13]. These studies and meta-analyses have also documented that the neuroadrenergic overactivity is already manifest in the prehypertensive state and becomes progressively more pronounced, as the severity of the blood pressure elevation becomes more marked [2,4–6,9–13]. This process will follow the chain of events, which participate at the cardiovascular continuum, favoring the progression of the disease, the occurrence of cardiovascular complications and the development of the hypertension-related target organ damage (Fig. 1). Both pharmacological and nonpharmacological blood pressure lowering interventions have been shown to exert, along with their antihypertensive properties, additional favorable effects known as “ancillary properties.” They may occur throughout a variety of mechanisms, including the drug-related sympathomodulatory properties aimed at reducing the degree of sympathoexcitation almost invariably characterizing the essential hypertensive state.

The present study, which is based on the European Society of Hypertension (ESH) Presidential Lecture given at the 32^nd^ ESH Meeting in Milan, will be focused on the effects of different nonpharmacological, pharmacological and devices-based interventions on the hypertension-related sympathetic overactivity. Following an introductory paragraph focused on the rationale for sympathomodulatory interventions in antihypertensive treatment, the most critical methodological aspects of the published studies aimed at assessing the antisympathetic therapeutic approaches adopted in the disease will be reviewed. The core of the study will be focused on the effects of nonpharmacological, pharmacological (monotherapy, combination drug treatment, new drugs), as well as devices-based interventions (renal sympathetic nerves ablation and carotid baroreceptor activation therapy) on the hypertension-related sympathetic overdrive. The final part of the manuscript will offer to the readers an outlook on the open questions related to this area of research that will need to be addressed in the forthcoming years. These include, among others, the new potential methodologies to assess sympathetic function during antihypertensive treatment and the link between adrenergic overdrive, adherence to antihypertensive treatment and controlled or uncontrolled blood pressure status.

## RATIONALE FOR SYMPATHOMODULATION IN HYPERTENSION TREATMENT

Recent years have seen a relevant increase in our know-how on the role of the sympathetic nervous system in hypertension pathophysiology and, more in general, in the determination of cardiovascular risk. The consistent evidence collected can be summarized as follows. First, different methodological approaches to investigate human sympathetic neural function have shown that a state of sympathetic overactivity characterizes the hypertensive condition, being detectable in the very initial hypertensive stages and becoming more and more pronounced, as the severity of the hypertensive state results more and more evident [1–13]. The relationship between blood pressure values and sympathetic overactivity is strengthened by the significant independent direct relationship detectable between these two variables in different studies and in a recent meta-analysis including data collected in more than 1000 hypertensive patients [5,6,9,12,13]. Second, the neuroadrenergic activation not only concurs at the blood pressure elevation, but it is also involved in favoring, along with the contribution of hemodynamic variables and other humoral factors, the development and progression of target organ damage, particularly at the level of the heart, the kidneys, the macro-circulation and the micro-circulation as well [12,14–17]. Of special interest is the participation of sympathetic factors to the occurrence of the endothelial dysfunction, which represents an early vascular/systemic alteration typical of the hypertensive state [18]. Third, metabolic alterations frequently detected in hypertensive patients, such as glucose intolerance, insulin resistance, hypertriglyceridemia, hypercholesterolemia and hyperuricemia, may be facilitated in their development by the adrenergic overdrive, particularly when prediabetes, overweight, metabolic syndrome, obesity and diabetes are detected as comorbidities of the hypertensive state [10,12]. Finally, an increased neuroadrenergic drive to the heart and the peripheral circulation has been shown to be associated with an augmented risk of future nonfatal and fatal cardiovascular events. The adverse prognostic impact of an elevated sympathetic cardiovascular drive, with close relationships with fatal events, has been documented in a variety of disease, such as chronic heart failure, acute thrombolembolic stroke, myocardial infarction, hepatic cirrhosis and end-stage renal failure, but not in hypertension [19–24]. In the hypertensive state, however, evidence exists that an indirect sympathetic marker such as an elevated heart rate value assessed at rest [25] may predict the future development of fatal and nonfatal cardiovascular events [26].

Altogether, the above-mentioned adverse consequences of the hypertension-related sympathetic activation represent a strong rationale for adopting in the treatment of this disease therapeutic approaches capable to modulate the adrenergic overdrive.

## METHODOLOGICAL DRAWBACKS OF THE PUBLISHED STUDIES

A significant number of published studies investigating the sympathetic effects of therapeutic interventions in hypertension suffer from intrinsic methodological limitations, which are summarized in Table 1. Four leading drawbacks will be worthy to be discussed here. First, in some studies, the effects of a given drug on sympathetic tone have been assessed after acute administration of the compound. In this case, a paradoxical increase in sympathetic tone has been almost invariably reported, which vanished when the evaluation with the same drug was done on a chronic basis, namely after days or weeks of regular daily treatment. This was the case even when the drug tested was a central sympatholytic agent [27]. The explanation for this paradoxical response is that the acute blood pressure reduction induced by the administration of a single dose of the antihypertensive agent tested triggers, almost invariably and independently on the pharmacological properties of the drug, a reflex sympathetic activation. This response appears to be largely unavoidable in its occurrence, because of the inability of the arterial baroreflex, which physiologically modulates sympathetic cardiovascular drive, to undergo an effective downward resetting before chronic treatment is established [28]. It should be emphasized that this limitation does apply not only to the studies investigating antihypertensive drugs, but also to the investigations aimed at exploring the effects of nonpharmacological or devices-based interventions. This is the case, for example, for the evaluation of the acute effects of physical exercise training on the adrenergic nervous system, which appear to be remarkably different from those reported during a physical training program prolonged for weeks or months [29,30]. This is also the case for renal denervation, whose sympathetic effects are different according to the time of performing the investigation after the procedure (days vs. weeks or vs. months) [31]. The second limitation refers to the fact that not rarely the studies have been carried out in normotensive individuals, which are characterized by a sympathetic function, which is already normal and thus impossible to be antagonized for obtaining an already present normalization. A further limitation is represented by the evidence that in the vast majority of the published studies the data on the blood pressure lowering effects of the therapeutic interventions were based on office rather than on 24-h blood pressure measurement, with all the well known limitations of this approach.

**TABLE 1:** Methodological limitations of published studies

• Intrinsic limitations of various indirect techniques to assess sympathetic function.
• Difficulties in assessing directly human sympathetic cardiovascular drive.
• Study population frequently including normotensive individuals.
• Lack of placebo-control group.
• Different pharmacokinetic profile of various drugs tested.
• Evaluation after acute drug administration.
• Blood pressure lowering effects frequently assessed on clinic rather than on 24-h ambulatory blood pressure measurements.
• Study population small.

Finally, a critical issue refers to the method used for assessing the sympathomoderating properties of a given therapeutic intervention. All the techniques available, which include the assay of the circulating venous plasma levels of the adrenergic neurotransmitter norepinephrine, the norepinephrine spillover technique, the power spectral analysis of the heart rate signal in specific bands as well as the microneurographic recording of efferent postganglionic sympathetic nerve traffic, display advantages and limitations already highlighted in previous studies [10,12,32]. Combination of two techniques to assess adrenergic function may allow to overcome some of these limitations, although the complexity of the investigation raises difficulties in repeating it various times before and during the treatment phase.

## SYMPATHOMODULATORY EFFECTS OF NON-PHARMACOLOGICAL INTERVENTIONS

The three main nonpharmacological approaches adopted in current clinical practice in the treatment of hypertension for which extensive information have been collected for their effects on sympathetic function include dietary restriction of salt intake, regular physical exercise and body weight reduction [33]. An additional nonpharmacological intervention, which is employed in specific clinical conditions characterized by the presence of the sleep apnea syndrome, is continuous positive airway pressure [34]. All these procedures display well defined sympathoinhibitory effects, the exception being represented by dietary restriction of salt intake, even when of moderate degree (80 mmol NaCl/day). Indeed, different methodological approaches employed to assess sympathetic function have almost univocally shown an increase in sympathetic cardiovascular drive during long-term dietary restriction of sodium intake. This is the case for the circulating venous plasma levels of the adrenergic neurotransmitter norepinephrine, renal norepinephrine spillover and muscle sympathetic nerve traffic, the increase in these sympathetic markers during sodium restriction averaging to 25–30% of the baseline prediet values [35–38]. Several mechanisms are likely to participate in the sodium restriction-related sympathoexcitation. These include an impairment of the inhibitory effects exerted by the arterial baroreceptors on the adrenergic function [38] and the development of an insulin resistance state, the resulting hyperinsulinemia triggering a marked increase in sympathetic cardiovascular drive [39,40].

As far as physical exercise training is concerned, there is evidence (although not always homogeneous) that the procedure may not only exert blood pressure lowering effects but also sympathomodulatory properties. These have been documented via the heart rate variability approach [41] and the regional norepinephrine spillover technique [42]. They have been more recently confirmed by microneurographic recording of muscle sympathetic nerve traffic, with an average reduction in the resting values approaching 40% [30,43]. The sympathoinhibitory effects, when evaluated via the microneurographic technique, are quite similar for magnitude to the ones detected in heart failure, according to the data presented in a recent meta-analysis [44].

Of a similar consistent nature are the results of the studies investigating the effects of body weight reduction obtained via dietary interventions alone or associated with bariatric surgery. Both the procedures induce a reduction in body weight, whose magnitude is extremely variable between studies and it was less, as expected, for the dietary interventions than for surgery [33]. The sympathoinhibitory properties of these interventions have been documented by different methoodological approaches to evaluate human adrenergic function, including the norepinephrine radiolabeled technique and the microneurographic recording of muscle sympathetic nerve traffic [45–48]. An analysis of the results of the microneurographic studies based on dietary interventions shows, together with a body weight reduction, a decrease in sympathetic neural outflow to muscle circulation amounting on average to 25%. This was associated with an office blood pressure lowering effect, which achieved statistical significance, however, for the SBP component only.

Finally, also the sympathoinhibitory effects of continuous positive airway pressure have been documented by different methodologies. The results of eight microneurographic studies with longitudinal design have been very recently included in a meta-analysis, showing a reduction amounting on average to about 25% of the baseline preintervention values [49]. The sympathoinhibition was associated with an office blood pressure reduction of quite consistent magnitude, that is, about 10.0 mmHg for systolic and 7.0 mmHg for diastolic [49].

## SYMPATHOMODULATORY EFFECTS OF PHARMACOLOGICAL INTERVENTIONS

### Monotherapy

All the five classes of the antihypertensive drugs recommended by current guidelines for antihypertensive treatment [33] have been extensively evaluated as monotherapies for their effects on sympathetic activity. The results can be outlined as follows. Beta-adrenergic blockers exert their blood pressure lowering effects by inducing cardiac and peripheral sympathetic modulation, which usually translates in clinical practice in a reduction of resting heart rate together with a peripheral vasodilation (particularly evident with beta-adrenergic blocking agents without sympathomimetic activity) [33,50]. The majority of the studies performed with these compounds evaluated the sympathetic effects only indirectly, such as measuring heart rate or circulating plasma levels of the adrenergic neurotransmitter norepinephrine. The few studies done with direct recording of efferent postganglionic sympathetic nerve traffic not always have shown, as should be expected from the pharmacological and clinical profile of the drug class, a marked sympathoinhibition, at variance from what has been reported in chronic heart failure [51]. It is likely that these discrepant results depend on the different levels of resting adrenergic overdrive characterizing these two conditions, which is more consistent in heart failure than in hypertension [51]. In contrast, a more effective degree of sympathoinhibition has been reported with drugs acting on the renin-angiotensin system, such as ACE-inhibitors and angiotensin II receptors blockers [52]. The data collected so far strongly support the notion that ACE-inhibitors and angiotensin II receptor antagonists reduce central sympathetic neural discharge in essential hypertensive individuals and may also cause inhibitory effects at the level of peripheral nerve terminals, modulating the spillover rate of the adrenergic neurotransmitter and improving its tissue clearance [52]. The drugs may also improve both vagal and sympathetic baroreflex control of the cardiovascular system, partially restoring this homeostatic function in treated hypertensive individuals [52]. Similar sympathoinhibitory effects have been documented for the renin inhibitor aliskiren [53].

Considering their sympathetic effects, calcium antagonists represent a heterogeneous group of compounds. Short-acting dihydropyridines exert clearcut sympathoexcitatory effects (likely dependent on a baroreflex impairment), which are reflected by the marked increase in heart rate, low-frequency component of heart rate variability, venous plasma norepinephrine and muscle sympathetic nerve traffic values detected following administration of these compounds [52]. These effects are hampered in the case of long-acting dihydropyridines, which however may display remarkable differences between different drugs belonging to the same pharmacological class [52].

Diuretic agents, particularly chlortalidone at a daily dosage greater than 25 mg, have been shown to elicit an increase in sympathetic drive, the magnitude of the occurring sympathoexcitation being however much less evident with chlorothiazide and indapamide. Along with volume-dependent sympathetic effects, diuretics may favor the occurrence of hyperinsulinemia, a metabolic alteration which, as already mentioned, may favor an increase in adrenergic cardiovascular drive [53]. In contrast, potent sympathoinhibitory effects have been described with the antialdosterone drug spironolactone, presumably dependent on baroreflex mechanisms [54].

### Old and new drugs

Clonidine, moxonidine and rilmenidine represent old and relatively new central sympatholytic drugs capable to inhibit various markers of adrenergic function, such as plasma norepinephrine, norepinephrine spillover and sympathetic nerve traffic [52]. As it will be mentioned in the following paragraph, central agents are now preferably used in the treatment of hypertension only as third or fourth agent in combination with other drugs [33]. In this case, the data available suggest that they can be of help in normalizing sympathetic neural function of the treated hypertensive patients [55]. Another class of drugs, statins, have been shown to exert significant sympathoinhibition [56], as also documented by a recent meta-analysis, which included five studies for a total of more than 80 patients [57]. The average reduction in sympathetic nerve traffic amounted to about 15% of baseline values [57]. The statins-related sympathoinhibition may depend on the improvement of baroreflex modulation of adrenergic drive described with these compounds [58] and may be responsible for the slight blood pressure reduction reported in recent studies and meta-analyses [59].

Two new classes of compounds which have been recently introduced in the therapeutic approach to cardiovascular and metabolic disease have been shown to exert modulatory effects on sympathetic neural function. These include sodium glucose co-transport protein-2 inhibitors and angiotensin receptor neprilysin inhibitors, which have been successfully employed in major clinical trials in the treatment of diabetic patients and patients with congestive heart failure, respectively. As far as the first group of compounds, conclusive evidence exists that they trigger favorable effects on glucose metabolism and blood pressure, which are associated with no change or even a reduction in different sympathetic markers [60,61]. The neprilysin inhibitor sacubitril, in association with an angiotensin ii receptor antagonist (valsartan), has been shown in chronic heart failure patients with a reduced left ventricular ejection fraction to be associated with an incidence of cardiovascular complications and events significantly lower than the one detected in the patients displaying a superimposable severity of the disease and under standard drug treatment without sacubitril [62]. These effects are accompanied by consistent sympathoinhibitory effects, as documented by the significant reduction (-18.0% on average) in muscle sympathetic nerve traffic values observed during prolonged administration of the drug [63].

### Combination drug treatment

The vast majority of the studies aimed at providing information on the sympathomodulatory effects of antihypertensive drug combinations have been based on indirect methods to assess the sympathetic responses to a given therapeutic intervention, namely the assay of the venous plasma levels of norepinephrine and the power spectral analysis of the heart rate signal. As discussed in previous studies [12,32], both the approaches have major limitations. In the case of plasma norepinephrine assay, the fact that the vasodilating properties of antihypertensive agents may reduce plasma norepinephrine levels increasing the tissue clearance of the sympathetic neurotransmitter [64,65]. This may produce misleading information on the impact of the drugs on sympathetic drive, wrongly pointing toward a sympathomodulation. In the case of the power spectral approach, it should be emphasized that the technique provides information only on sympathetic/parasympathetic balance at the level of the heart, which frequently does not reflect the effects of a given therapeutic intervention on systemic sympathetic neural function [12,32].

**FIGURE 2:**
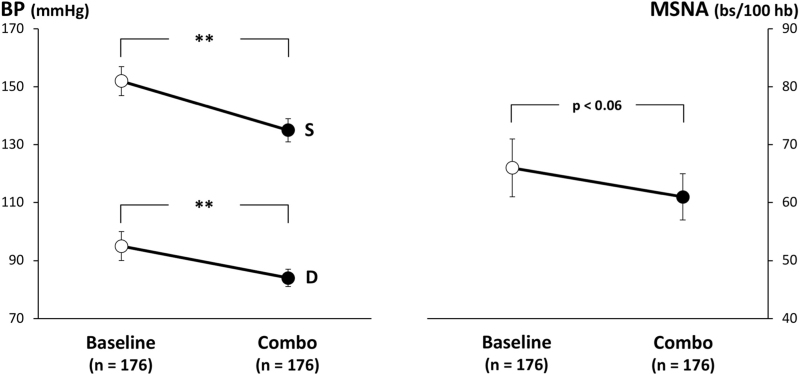
Effects of two antihypertensive drugs combinations on SBP (S), DBP (D) and muscle sympathetic nerve traffic (MSNA) in 176 patients enrolled in different microneurographic studies. Data are shown as means ± SD. Asterisks (^∗∗^*P* *<* *0.01*) refer to the level of statistical significance between values recorded before (baseline) and during combination drugs treatment (combo).

An analysis of the results of the studies aimed at assessing the sympathetic responses to therapeutic interventions via the direct microneurographic recording of sympathetic nerve traffic has provided the following information. Results of the nine studies enrolling on the whole more than 170 hypertensive patients under combination treatment have shown a consistent significant blood pressure reduction (17.0 mmHg for systolic and 11.0 mmHg for diastolic) coupled with a reduction in sympathetic nerve traffic (10–15% of baseline values) close to achieve statistical significance (Fig. 2). The effects on the sympathetic function were more marked when the drugs combination was based on an ACE-inhibitor and an angiotensin II receptor blocker or a central sympatholytic agent.

As a general rule, with the exception of the study mentioned above, which made use of the combination treatment between an angiotensin II receptor blocker and a central sympatholytic agent [55], no published study reported during treatment a reduction in sympathetic nerve traffic at values similar to the ones detected in pure normotensive control individuals. The lack of a full sympathetic normalization during antihypertensive drug treatment has been hypothesized as to be one of the mechanisms responsible for the so-called ‘residual risk’ [66], that is, for the fact that blood pressure lowering drugs, despite reducing blood pressure to normal values, do not allow to bring back to ‘normal’ the cardiovascular risk of the treated hypertensive patient [67].

## SYMPATHOMODULATORY EFFECTS OF DEVICES-BASED INTERVENTIONS

**FIGURE 3:**
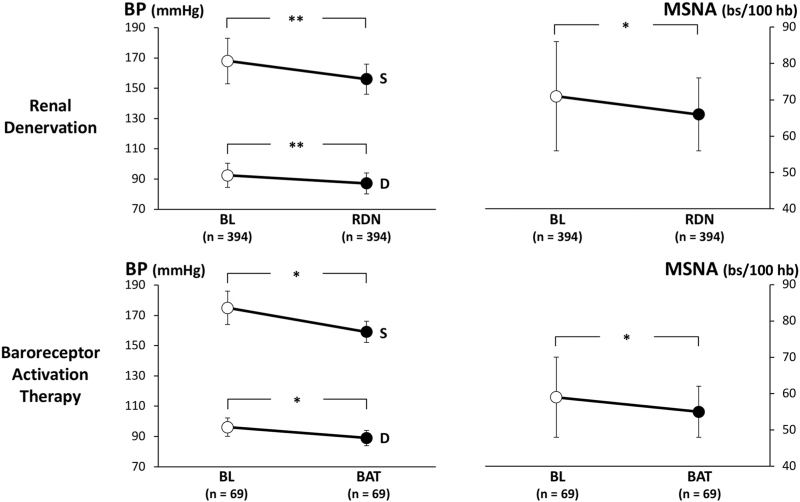
Effects of bilateral renal nerves ablation (RDN, upper) and carotid baroreceptor activation therapy (BAT, lower) on SBP (S), DBP (D) and muscle sympathetic nerve traffic (MSNA) in patients enrolled in different microneurographic studies. Data are shown as means ± SD. Asterisks (^∗∗^*P* *<* 0.01, ^*∗*^* P* *<* 0.05) refer to the level of statistical significance between values recorded before (BL) and during RDN or BAT treatment.

There are two main assumptions supporting the analysis of the effects of devices-based interventions on sympathetic cardiovascular drive. The first one is represented by the evidence that drug-resistant hypertension, that is, the hypertensive phenotype identified by earlier studies as preferred target of the devices-based interventions, such as bilateral renal nerve ablation and carotid baroreceptor activation therapy, is characterized by a remarkable sympathetic overdrive [11,13]. The second assumption, on the contrary, is that the sympathetic activation is responsible for the development of the resistant hypertensive state, its reduction by devices being associated with a clearcut decrease in blood pressure values [67]. The two assumptions have received throughout the years experimental and clinical supports, although not always homogeneous in their nature, however. Two main successful approaches have been used, that is, the radiofrequency or, more recently, endovascular ultrasound bilateral ablation of renal nerves and carotid baroreceptor activation therapy [68]. In a recent meta-analysis done by our group including 11 microneurographic studies published so far for a total of about 400 patients, bilateral renal nerves ablation elicited a blood pressure lowering effect associated with a reduction in muscle sympathetic nerve traffic at the sixth month follow-up (Fig. 3, upper) [69]. Interestingly, the blood pressure and the sympathetic effects do not appear to follow the same time course; however, the occurrence of the blood pressure reduction preceding by weeks the sympathetic one [31]. In addition, the blood pressure reduction and the sympathoinhibition did not show any significant quantitative relationships among each other [68]. The hypothesis has been therefore advanced that the blood pressure lowering effects of the procedure may be driven by extrasympathetic mechanisms and not necessarily only by the sympathetic ones [69].

Figure 3, lower, illustrates the effects of carotid baroreceptor activation therapy obtained in four studies on blood pressure and sympathetic neural drive. Similar to what is described in the case of renal nerves ablation, also in this case, the procedure caused a significant blood pressure reduction combined with a sympathoinhibition. However, at variance from renal denervation, the magnitude of the blood pressure effect was quantitatively and often qualitatively related to the degree of the sympathetic inhibition [70]. As a final consideration of the analysis of the data obtained with renal nerves ablation and carotid baroreceptor activation therapy, it should be worthy to mention that, similarly to what has been already described for combination drug treatment, both the procedures, although effective in reducing elevated sympathetic nerve traffic values, fail to obtain their full normalization.

## OPEN QUESTIONS AND CONCLUSION

Results of recent studies have allowed to prompt the hypothesis that the clinical condition characterized by uncontrolled blood pressure values may be linked with, and possibly dependent on, an elevated degree of sympathetic activation [70–72]. This alteration would prevent antihypertensive drug treatment to achieve blood pressure control, thus determining the clinical phenotype defined as ‘refractory hypertension.’ It should be mentioned that as these studies were based on indirect (and frequently inadequate) methods for assessing sympathetic cardiovascular drive, future investigations assessing directly adrenergic drive via the microneurographic technique should be planned. A further question which is still unanswered is whether and to what extent adherence to treatment is related to sympathetic activation. In other words, it is unknown whether hypertensive patients who display a poor compliance to treatment are characterized by a sympathetic overactivity greater for magnitude than the one detected in patients, which display a full treatment adherence.

Finally, in a next future, the investigation of the sympathomodulatory effects of nonpharmacological, pharmacological or device-based antihypertensive treatment should be simplified by the use of techniques capable to assess neuroadrenergic function in a less complex fashion. In this context, promising approaches are represented by the assessment of skin blood flow responses to antihypertensive treatment during mental stress via a tissue flowmeter [73]. Another approach, already tested in some studies investigating the effects of calcium channel blockers on adrenergic cardiovascular drive, is pupillometry, assessing pupillary diameters changes induced by light stimulus [74]. These new approaches to assess human sympathetic function, once validated and adequately tested for their main features, will hopefully allow to collect information on the sympathomodulatory effects of different therapeutic interventions adopted in hypertension treatment in large-scale clinical studies. Indeed, it should be emphasized that the information collected so far on this issue are obtained in small studies. This represents an important limitation, making it difficult to extrapolate the data collected in small groups of hypertensive patients to the large hypertensive population.

## ACKNOWLEDGEMENTS

### Conflicts of interest

There are no conflicts of interest.
